# Clinical Efficacy on Fracture Risk and Safety of 0.5 mg or 1 mg/month Intravenous Ibandronate Versus 2.5 mg/day Oral Risedronate in Patients with Primary Osteoporosis

**DOI:** 10.1007/s00223-013-9734-6

**Published:** 2013-05-05

**Authors:** Toshitaka Nakamura, Tetsuo Nakano, Masako Ito, Hiroshi Hagino, Junko Hashimoto, Masato Tobinai, Hideki Mizunuma, For the MOVER Study Group

**Affiliations:** 1 Department of Orthopedic Surgery, University of Occupational and Environmental Health, 1-1 Iseigaoka, Yahata-nishi-ku, Kitakyushu, Fukuoka, 807-8555 Japan; 2 Tamana Central Hospital, Kumamoto, Japan; 3 Medical Work-Life Balance Center, Nagasaki University Hospital, Nagasaki, Japan; 4 School of Health Science & Rehabilitation Division, Faculty of Medicine, Tottori University, Tottori, Japan; 5 Project & Life Cycle Management Unit, Chugai Pharmaceutical Co. Ltd., Tokyo, Japan; 6 Clinical Development Division, Chugai Pharmaceutical Co. Ltd., Tokyo, Japan; 7 Department of Obstetrics and Gynecology, Hirosaki University School of Medicine, Hirosaki, Japan

**Keywords:** Ibandronate, Intravenous, Osteoporosis, Risedronate, Vertebral fracture

## Abstract

This randomized, double-blind study assessed the antifracture efficacy and safety of intermittent intravenous (IV) ibandronate versus oral daily risedronate in Japanese patients with primary osteoporosis. Ambulatory patients aged ≥60 years were randomized to receive 0.5 or 1 mg/month IV ibandronate plus oral daily placebo or 2.5 mg/day oral risedronate, the licensed dose in Japan, plus IV placebo. The primary end point was noninferiority of ibandronate versus risedronate for first new or worsening vertebral fracture over 3 years. A total of 1,265 patients were randomized. A total of 1,134 patients formed the per-protocol set. Both ibandronate doses were noninferior to risedronate: 0.5 mg, hazard ratio (HR) 1.09 [95 % confidence interval (CI) 0.77–1.54]; 1 mg, HR 0.88 (95 % CI 0.61–1.27). The rate of first new vertebral fracture over 3 years was 16.8 % (95 % CI 12.8–20.8) for 0.5 mg ibandronate, 11.6 % (95 % CI 8.2–15.0) for 1 mg ibandronate, and 13.2 % (95 % CI 9.6–16.9) for risedronate. Significant increases in bone mineral density relative to baseline were observed with all treatments after 6 months, with substantial reductions in bone turnover markers after 3 months. Greatest efficacy was obtained with 1 mg ibandronate. Analyses in women only showed similar results to the overall population. No new safety concerns were identified. This study demonstrated the noninferiority of IV ibandronate to the licensed Japanese dose of oral risedronate and suggested that 1 mg/month is an effective dose in Japanese patients with primary osteoporosis.

The efficacy of ibandronate on vertebral fractures has been demonstrated in postmenopausal osteoporosis [1, 2]. In the randomized, double-blind trial BONE (oral iBandronate Osteoporosis vertebral fracture trial in North America and Europe), oral ibandronate 2.5 mg/day or 20 mg every other day for 12 doses every 3 months significantly reduced the risk of vertebral fracture and increased bone mineral density (BMD) at the lumbar spine and total hip versus placebo [3]. Two further randomized studies, MOBILE (Monthly Oral iBandronate In LadiEs) [2, 4–6] and DIVA (Dosing IntraVenous Administration) [1, 7–9], confirmed the noninferiority of BMD gains in the lumbar spine with 100 and 150 mg once-monthly tablets or 2 mg/2 months or 3 mg/3 months intravenous (IV) injections to those with daily oral ibandronate 2.5 mg for up to 5 years. The efficacy of ibandronate in increasing BMD in male osteoporosis patients has also been reported [10].

Three large randomized studies have demonstrated the efficacy of risedronate against vertebral, nonvertebral, and hip fractures [11–13]. Compared with placebo, oral risedronate 2.5 or 5 mg daily significantly reduced the relative risk of hip fracture among elderly women with confirmed osteoporosis in the HIP (Hip Intervention Program) study, with relative risks of hip fracture of 0.5 and 0.7, respectively [11]. Oral risedronate (2.5 or 5 mg daily) also significantly reduced the risk of new vertebral fracture by 46 and 65 %, respectively, compared with placebo in the VERT-NA (Vertebral Efficacy with Risedronate Therapy North America) study, and reduced the fracture risk by similar amounts in postmenopausal women with prevalent vertebral fractures in the VERT-MN (VERT-Multinational) study [14]. The plasma concentrations of risedronate attained while on treatment with 2.5 mg oral risedronate in Japanese subjects were almost comparable with those of 5 mg dosing in white subjects [15]. Furthermore, BMD increases at the lumbar spine and changes in bone turnover markers (BTMs) were comparable in Japanese subjects who received 2.5 mg/day risedronate and in white patients who received 5 mg/day risedronate [13, 16]. In a dose-ranging study of risedronate in Japanese patients with osteoporosis, a linear dose–response relationship for increases in BMD and decreases in BTMs was obtained up to a dose of 2.5 mg, but no further increase was observed with 5 mg risedronate [17]. Based on these results, the optimal oral dose of risedronate in Japanese osteoporotic patients was determined and licensed as 2.5 mg daily or 17.5 mg weekly [18]. Reduction in fracture risk has been observed for vertebral [19] and hip [20] fractures at this dose. Although placebo-controlled data for fracture risk have not been obtained in Japanese patients, risedronate 2.5 mg daily is a suitable active comparator to assess fracture prevention efficacy in Japanese osteoporotic patients.

European and North American patients enrolled in BONE achieved comparable efficacy with ibandronate [21], but the effect on osteoporosis fracture risk has not been well investigated in nonwhite patients. In a randomized study in Japanese women with postmenopausal osteoporosis, IV ibandronate 0.5 mg/month, 1 mg/month and 2 mg/2 months substantially increased lumbar spine BMD and significantly reduced BTMs compared with placebo [22]. Meta-analyses have shown that fracture risks of men and women are similar for any given BMD [23, 24]; thus postmenopausal osteoporosis and osteoporosis in elderly men can be categorized as primary osteoporosis with possible heterogeneous pathogenesis [25, 26]. The current study was conducted for registration purposes in Japan and evaluated the efficacy and safety of IV ibandronate 0.5 mg and 1 mg/month versus oral daily risedronate 2.5 mg (the licensed dose in Japan) in terms of vertebral fracture incidence in patients with osteoporosis, including both postmenopausal women and older men.

## Materials and Methods

### Study Design

The MOVER (MOnthly intraVenous ibandronatE versus daily oral Risedronate) study was a prospective, randomized, double-blind, active drug-controlled study comparing IV ibandronate (0.5 mg and 1 mg/month) with 2.5 mg/day oral risedronate over 3 years in women and men (ClinicalTrials.gov identifier: NCT00447915). The primary end point was noninferiority of ibandronate versus risedronate with regards to the incidence of nontraumatic morphometric vertebral fractures at 3 years. Institutional review boards from the participating centers provided ethical approval and the study was conducted in accordance with the Declaration of Helsinki and the International Conference on Harmonization of Good Clinical Practice Guidelines. All patients provided written informed consent prior to any study-related procedure.

### Patients

Ambulatory women or men aged ≥60 years with primary osteoporosis according to the Diagnosis Criteria of Primary Osteoporosis in Japan [27] were eligible if they had: fragile bone fracture (nontraumatic osteoporotic fracture that occurred by slight external force combined with low BMD); BMD of the lumbar spine (L2–L4), or proximal femur (total hip and femoral neck) <80 % of the young adult mean (equivalent to *T* score–1.7,–1.6, and–1.4, respectively); and 1–5 radiographically confirmed vertebral fractures in the fourth thoracic spine–fourth lumbar spine (Th4–L4).

Exclusion criteria included: vertebral deformations likely to affect vertebral strength; previous radiotherapy of the thoracic spine/lumbar spine/pelvis; secondary osteoporosis or a disease causing decrease in bone volume; a disorder delaying the passage of food through the esophagus; received/planned invasive dental procedures; bisphosphonate use within 1 year of the start of the study, or prior treatment with ibandronate, anti-RANKL antibody (AMG162) or strontium; receipt of drugs likely to affect bone metabolism within 8 weeks of the start of the study; severe cardiac, renal or hepatic disease; calcium outside the criteria value (i.e., <8.4 mg/dL or >10.4 mg/dL); hypersensitivity to bisphosphonate, calcium or vitamin D; active malignant tumor or prior therapy for malignant tumor within 3 years.

### Treatment

Patients were randomly assigned to receive: 0.5 mg/month IV ibandronate (F. Hoffmann-La Roche, Ltd.) plus oral daily placebo for 36 months; 1 mg/month IV ibandronate plus oral daily placebo; or 2.5 mg/day oral risedronate (Ajinomoto Co. Inc.) plus IV placebo by the double dummy method. All patients received supplementary calcium 305 mg and vitamin D 200 IU/day [28] as a single tablet daily throughout the study period. IV study drug administration was recorded by the investigator at the time of dosing, while oral study drug administration was surveyed by the patient and recorded by the investigator. Based on published data comparing the efficacy and safety of 1 and 2 mg/2 months IV ibandronate in Japanese patients [22], the 1 mg/month dose was selected for the current study. As weekly risedronate was not marketed in Japan when the study was planned, and daily risedronate was the most popular bisphosphonate in use, daily oral risedronate was selected as the active comparator for this trial.

### Randomization and Blinding

Randomization was performed centrally through dynamic allocation (minimization method) based on the number of prevalent vertebral fractures (1 vs. >1). Patients, investigators, steering committee members, the sponsor, and the faculty who adjudicated the study end points remained unaware of treatment-group assignments throughout the trial.

### Study End Points

The primary end point was the incidence of nontraumatic morphometric vertebral fractures including new vertebral fractures and worsening of prevalent vertebral fractures at 3 years. Secondary end points were: the incidences of nontraumatic new vertebral fractures, all osteoporotic nonvertebral fractures, osteoporotic nonvertebral fractures at the six major sites (femur, forearm, humerus, clavicle, tibia/fibula, pelvis), clinical vertebral fractures, and total clinical fractures; percentage change from baseline in lumbar spine (L2–L4), total hip, trochanter and femoral neck BMD; change from baseline in BTMs of urinary C- and N-telopeptide of type 1 collagen corrected by creatinine (uCTX and uNTX, respectively), serum bone-specific alkaline phosphatase (BALP) and osteocalcin (OC); and safety.

### Schedule of Assessments

Radiographs of the thoracic and lumbar spine were taken at screening, baseline, and at 6, 12, 24, and 36 months after treatment for the assessment of fractures. To identify morphometric vertebral fractures, the vertebral bodies of the lateral projection from Th4 to L4 were assessed using semiquantitative (SQ) methodology and quantitative morphometry (QM) [29] by a central committee who were blinded to treatment. Prevalent fractures were defined as vertebrae with an anterior/posterior height ratio <0.75, or central/posterior height ratio <0.80, or a 20 % reduction in any of the anterior, posterior, or central vertebral heights from corresponding values in the adjacent upper or lower vertebra. A new vertebral fracture was defined as an increase of ≥1 SQ grading scale in a vertebra that was normal at baseline, while a worsening fracture was defined as an increase of ≥1 SQ grading scale in a vertebra that was deformed at baseline. Fracture incidence was adjudicated by three experts with reference to QM data from Synarc (San Francisco) and a binary SQ assessment was made. Radiographs were assessed to identify nonvertebral fractures in patients with clinical symptoms.

BMD measurements in lumbar spine (L2–L4), total hip, trochanter and femoral neck were performed centrally at screening, baseline, 6, 12, 24, and 36 months using dual-energy X-ray absorptiometry of Hologic and Lunar bone densitometers. BTMs were measured centrally at baseline, 3, 6, 12, 24, and 36 months. Urine samples were obtained under fasting conditions. All samples were collected prior to administration of injection.

Adverse events (AEs) were summarized throughout the study and for up to 15 days after study end. AEs of interest such as renal, cardiac and gastrointestinal functions, acute phase reactions (APRs), hypocalcemia, osteonecrosis of the jaw, and atypical fracture of the femur, were specified in advance.

### Statistical Analyses

The primary analysis was performed on the per-protocol set (PPS). For the analysis of vertebral fracture incidence, stratified Cox regression with number of prevalent vertebral fractures (1 vs. ≥2) and age (60–74 vs. ≥75 years) as stratified variables and life table method was used. The log rank test was applied for between-treatment group comparisons of fracture incidence. To control the overall significance level, a closed testing procedure from higher dose was applied. Noninferiority of IV ibandronate to oral risedronate would be concluded if the upper limit of the 90 % confidence interval (CI) of the hazard ratio (HR) was below the confidence limit of noninferiority of 1.55.

Analyses of BMD and BTMs were based on the relative change from baseline and between-treatment group comparisons were performed by *t* test. Missing data were imputed by the last observation carried forward method.

Based on published data [19], the fracture incidence rate of risedronate after 3 years was estimated to be 17 % and that of ibandronate to be 16 %. Under these assumptions, we calculated that 295 patients were required in each treatment group to assess the noninferiority of ibandronate to risedronate with the HR threshold value of 1.55. A one-sided significance level of 0.05 was set, with a detection power of 80 % by the Shoenfeld method. With an expected drop-out rate of 25 %, 394 patients were required in each treatment group, giving a total of 1,182 patients. The study was not designed to compare the two ibandronate doses; however, their significance was assessed as an exploratory measure.

## Results

### Patient Disposition and Baseline Characteristics

**Fig. 1:**
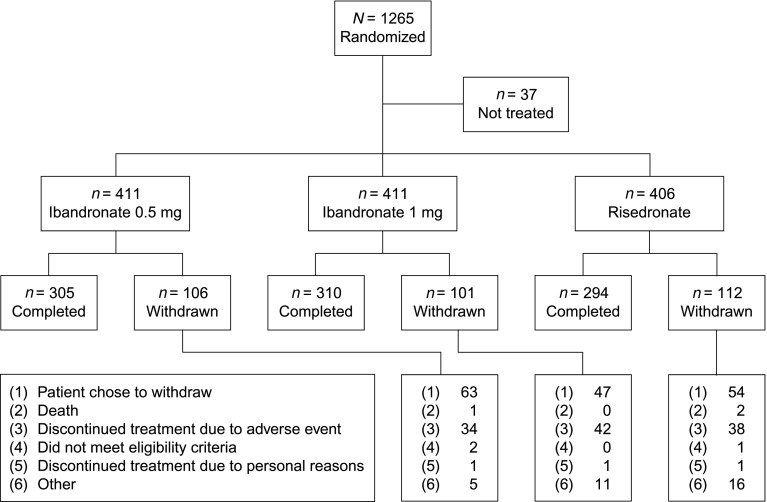
Patient flow through the study (men and women)

A total of 1,265 patients were randomized. Thirty-seven patients did not receive study treatment, leaving 1,228 patients in the safety population: 411, 411 and 406 patients (389, 381 and 371 women) were randomized to receive ibandronate 0.5 mg, ibandronate 1 mg, and risedronate, respectively (Fig. 1). Overall, 909 patients (854 women) completed the study. The PPS for the primary end point analysis comprised 1,134 patients, including 376, 382 and 376 (356, 354 and 343 women) in the ibandronate 0.5 mg, ibandronate 1 mg and risedronate groups, respectively (Table 1). Baseline patient characteristics were well balanced between the treatment groups.

**Table 1:** Baseline patient characteristics

Characteristic	Ibandronate 0.5 mg	Ibandronate 1 mg	Risedronate
	(*n* = 376)	(*n* = 382)	(*n* = 376)
Women, *n* (%)	356 (94.7)	354 (92.7)	343 (91.2)
Age (year), mean (SD)	72.9 (6.34)	72.2 (6.38)	73.0 (6.29)
Aged 60–74 year, *n* (%)	219 (58.2)	245 (64.1)	227 (60.4)
Aged ≥75 year, *n* (%)	157 (41.8)	137 (35.9)	149 (39.6)
Weight (kg), mean (SD)	50.6 (8.00)	50.9 (7.36)	51.1 (8.35)
Height (cm), mean (SD)	149.2 (6.66)	149.5 (6.56)	149.4 (6.70)
BMD *T*-score, mean (SD)			
Lumbar spine (L2–L4)	−2.71 (1.01)	−2.68 (1.01)	−2.59 (1.06)
Femoral neck	−2.48 (0.73)	−2.41 (0.80)	−2.53 (0.79)
Total hip	−2.17 (0.87)	−2.09 (0.86)	−2.18 (0.86)
Prevalent vertebral fractures, *n* (%)			
1	186 (49.5)	184 (48.2)	183 (48.7)
2	97 (25.8)	106 (27.7)	95 (25.3)
>2	93 (24.7)	92 (24.1)	98 (26.1)
uCTX (μg/mmol CR), mean (SD)	382.4 (226.2)	368.6 (209.9)	373.2 (261.0)
uNTX (nM BCE/mM CR), mean (SD)	73.6 (39.31)	69.4 (35.42)	68.9 (35.16)
BALP (IU/L), mean (SD)	33.6 (13.15)	33.9 (13.11)	32.4 (11.96)
25-OH vitamin D (ng/mL), mean (SD)	19.6 (6.44)	20.0 (6.69)	19.7 (6.56)

*BALP* bone-specific alkaline phosphatase, *BCE* bovine collagen equivalent, *BMD* bone mineral density, *CR* creatinine, *SD* standard deviation, *uCTX* creatinine-corrected urinary collagen type 1 cross-linked C-telopeptide, *uNTX* creatinine-corrected urinary collagen type 1 cross-linked N-telopeptide

The modified intent-to-treat (ITT) population of 1,220 patients included 404, 411 and 405 patients (382, 381 and 370 women) in the ibandronate 0.5 mg, ibandronate 1 mg, and risedronate groups, respectively. No difference in discontinuation rate was found between the groups: 25.8, 24.6 and 27.6 %, respectively. Compliance rates in the modified ITT population were >96 % for IV administration and >93 % for oral administration.

Mean 25-hydroxyvitamin D levels were low at baseline (Table 1), but increased above 25.0 ng/mL in all treatment groups after 3 years: 26.6 ng/mL [standard deviation (SD) 6.09], 26.6 ng/mL (SD 6.71), and 26.9 ng/mL (SD 6.07) in the ibandronate 0.5 mg, ibandronate 1 mg and risedronate groups, respectively.

### Incidence of Vertebral Fractures

**Fig. 2:**
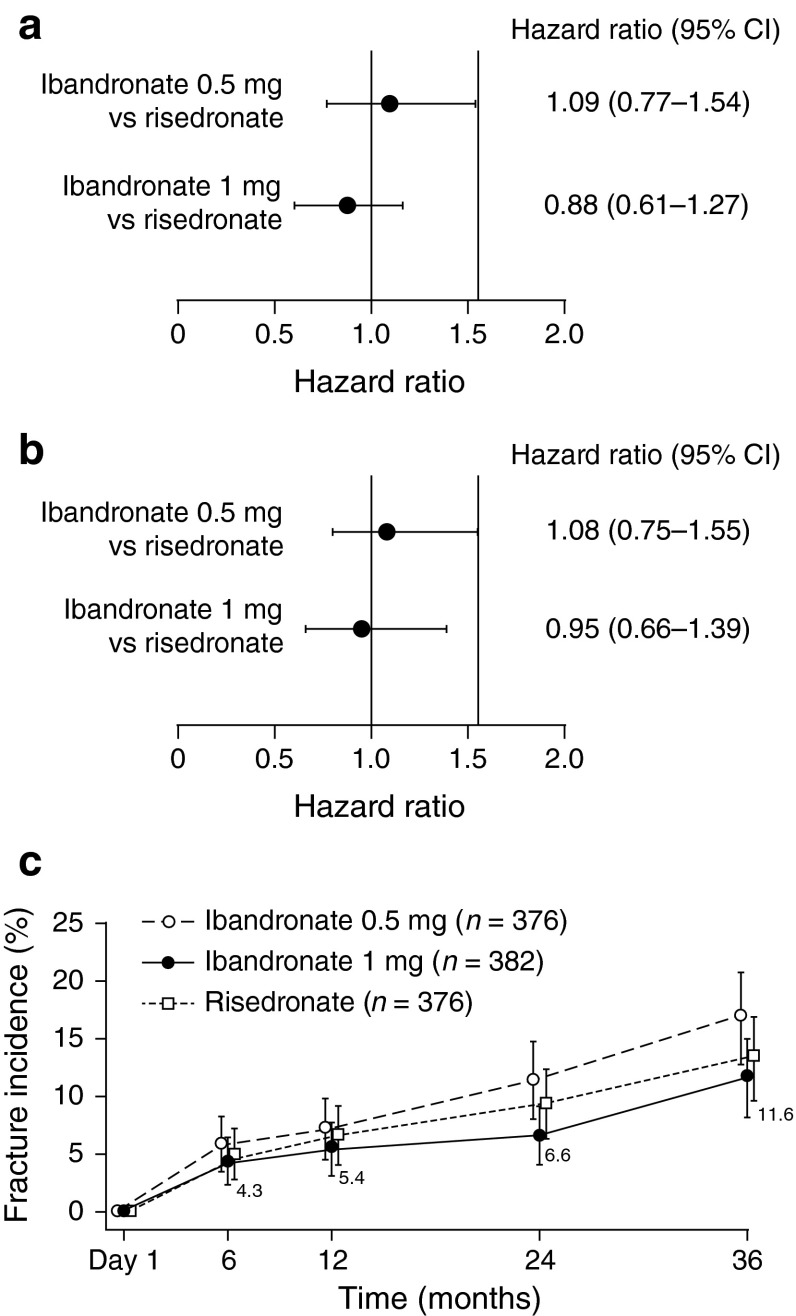
Vertebral fracture efficacy: forest plot of hazard ratios for the first new or worsening vertebral fracture in **a** all patients and **b** women only. **c** Life table analysis for the first new vertebral fractures during the study. *CI* confidence interval

There was no difference in the incidence of morphometric vertebral fractures at screening or at baseline. The cumulative incidences of new or worsening vertebral fractures over 3 years were 19.9 % (95 % CI 15.6–24.1), 16.1 % (95 % CI 12.2–19.9) and 17.6 % (95 % CI 13.6–21.6) for the ibandronate 0.5 mg, ibandronate 1 mg, and risedronate groups, respectively. Compared with the risedronate group, the HRs for fracture incidences were 1.09 (95 % CI 0.77–1.54) and 0.88 (95 % CI 0.61–1.27) for ibandronate 0.5 mg and 1 mg, respectively (Fig. 2a). The HRs for fracture incidences for women only were 1.08 (95 % CI 0.75–1.55) and 0.95 (95 % CI 0.66–1.39), respectively, over 3 years (Fig. 2b). At 2 years, the incidence of vertebral fractures in women only was 13.4 % (95 % CI 9.8–17.1), 11.0 % (95 % CI 7.6–14.3) and 12.8 % (95 % CI 9.1–16.4) for the ibandronate 0.5 mg, ibandronate 1 mg, and risedronate groups, respectively, with HRs of 1.01 (95 % CI 0.66–1.54) and 0.83 (95 % CI 0.53–1.29), respectively. At 1 year, the fracture incidence in women only was 9.6 % (95 % CI 6.5–12.7), 8.4 % (95 % CI 5.5–11.4) and 10.8 % (95 % CI 7.4–14.1), with respective HRs of 0.85 (95 % CI 0.53–1.37) and 0.76 (95 % CI 0.46–1.23).

The cumulative incidences of first new vertebral fractures were 16.8 % (95 % CI 12.8–20.8), 11.6 % (95 % CI 8.2–15.0), and 13.2 % (95 % CI 9.6–16.9) for the ibandronate 0.5 mg, ibandronate 1 mg, and risedronate groups, respectively (Fig. 2c). The HR for the ibandronate groups compared with the risedronate group were 1.27 (95 % CI 0.86–1.89) and 0.87 (95 % CI 0.57–1.33) for the 0.5 mg and 1 mg doses, respectively; the difference between the 0.5 mg and 1 mg ibandronate doses was not statistically significant (*P* = 0.062).

### Incidence of Osteoporotic Nonvertebral Fractures and Clinical Fractures

**Fig. 3:**
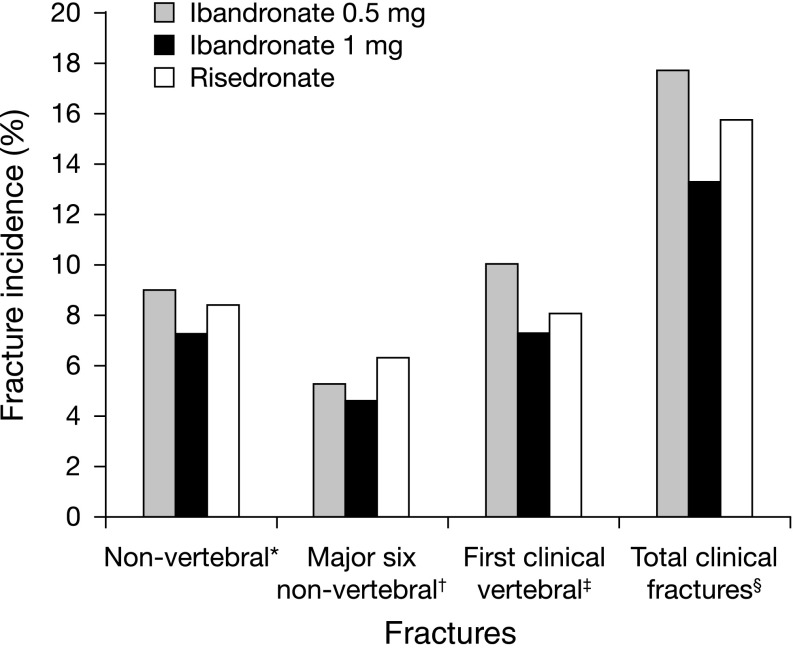
Incidences of osteoporotic nonvertebral fractures, major six nonvertebral fractures, first clinical vertebral fractures, and total clinical fractures through 3 years. *P* value (log rank) for ibandronate versus risedronate: *P *=* 0.652 (0.5 mg), *P* *=* 0.605 (1 mg) ^†^ *P* *=* 0.752 (0.5 mg), *P* *=* 0.449 (1 mg) ^‡^ *P* *=* 0.468 (0.5 mg), *P* *=* 0.568 (1 mg) ^§^ *P* *=* 0.497 (0.5 mg), *P* *=* 0.298 (1 mg)

The cumulative incidences of osteoporotic nonvertebral fractures were 9.0, 7.2, and 8.4 % for the ibandronate 0.5 mg, ibandronate 1 mg, and risedronate groups, respectively (Fig. 3). The difference between the ibandronate groups was not statistically significant. The respective values for the major six nonvertebral fractures were 5.3, 4.6, and 6.3 %. Differences between the treatment groups were not statistically significant for any of the fracture end points.

### Bone Mineral Density

**Fig. 4:**
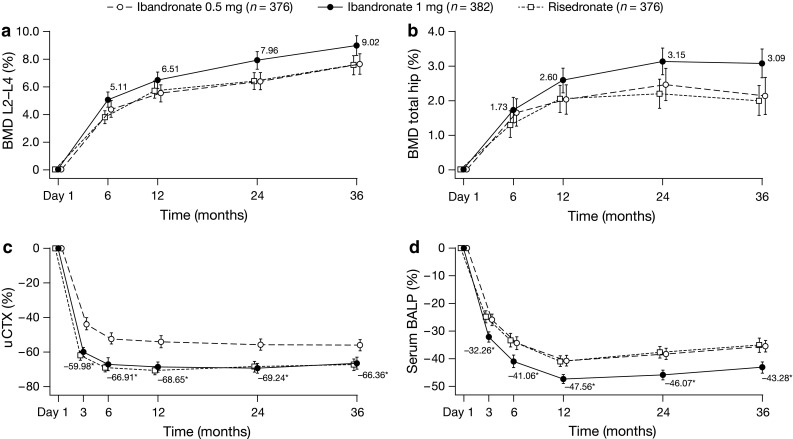
Mean relative change from baseline (with 95 % CI) throughout 3 years in **a** BMD at the lumbar spine (L2–L4); **b** BMD at the total hip; **c** uCTX; **d** serum BALP. *P* value (*t* test) for 1 mg ibandronate versus risedronate: L2–L4: *P* *=* 0.001 (6 months), *P* *=* 0.001 (24 months), *P* *=* 0.005 (36 months), total hip: *P* *=* 0.001 (24 months), *P* < 0.001 (36 months), *P < 0.005 for 1 mg ibandronate versus 0.5 mg ibandronate. *CI* confidence interval, *L* lumbar, *BMD* bone mineral density, *uCTX* creatinine-corrected urinary collagen type 1 cross-linked C-telopeptide, *BALP* bone-specific alkaline phosphatase

At 3 years, the mean relative change from baseline in BMD values for the ibandronate 0.5, 1 mg and risedronate groups was 7.7, 9.0, and 7.6 %, respectively, for the lumbar spine (Fig. 4a), and 2.2, 3.1, and 2.0 %, respectively, for the total hip (Fig. 4b). Respective values at the trochanter were 3.8, 4.7, and 3.1 %, and at the femoral neck were 2.1, 3.1, and 2.2 %. Significant differences were noted in lumbar spine BMD between the 1 mg and 0.5 mg ibandronate dose groups at 1 year (*P* *=* 0.030), 2 years (*P* *=* 0.001), and 3 years (*P* *=* 0.010), and in total hip BMD at 2 years (*P* *=* 0.029) and 3 years (*P* *=* 0.006). Intergroup differences in BMD between the ibandronate groups were not significant at the trochanter. BMD changes in women showed similar trends in all three treatment groups (data not shown).

### Bone Turnover Markers

The mean relative change from baseline in uCTX (Fig. 4c) and uNTX levels was similar with ibandronate 1 mg and risedronate, with an initial decrease at 3 months and levels maintained below baseline throughout the study. Mean reductions from baseline at 6 months with 1 mg ibandronate were 67 and 53 % for uCTX and uNTX, respectively. With ibandronate 0.5 mg, decreases in uCTX and uNTX were less than those in the other treatment groups, and there was no overlap between the 95 % CIs with either ibandronate 1 mg or risedronate.

In the ibandronate 1 mg group, serum BALP (Fig. 4d) and OC levels decreased at 3 months and remained below baseline thereafter; at 6 months, mean relative changes from baseline were 41 and 35 %, respectively. Decreases in serum BALP and OC levels in the ibandronate 0.5 mg and risedronate groups were less than those in the ibandronate 1 mg group and the 95 % CIs of the ibandronate 0.5 mg and risedronate groups did not overlap with those of the ibandronate 1 mg group. Significant differences were noted between the ibandronate groups at each time point for all BTMs (*P* < 0.005). Women showed similar changes as the overall population (data not shown).

### Adverse Events

**Fig. 5:**
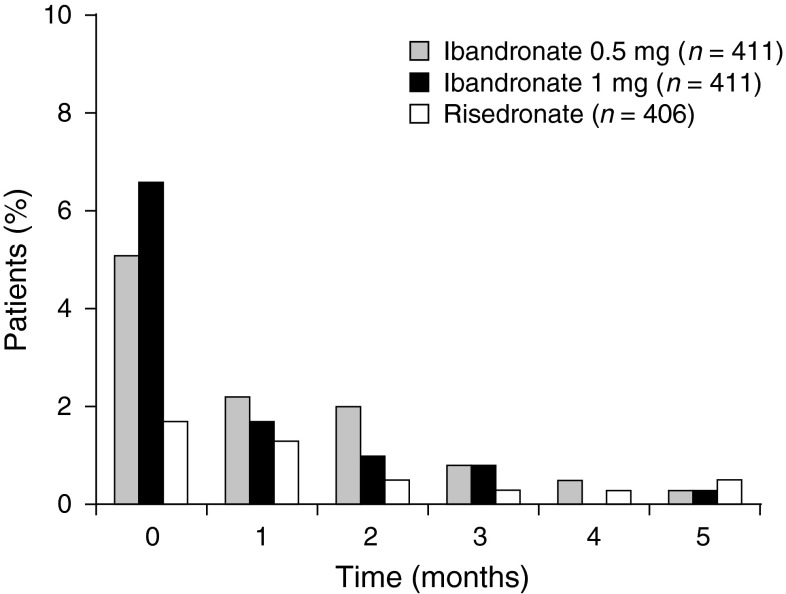
Change in the incidence of acute phase reactions between first and subsequent dose of study medication

No significant differences were observed between the treatment groups with respect to the incidence of all AEs, serious AEs, AEs leading to death or AEs leading to withdrawal (Table 2). Regarding AEs of interest, the most frequently reported renal-related AEs were increased blood creatinine and the presence of protein in urine. All renal function-related AEs were mild, and there were no significant differences in incidence between the groups (Table 3). Most APR AEs were mild in intensity and transient, and decreased with each subsequent dose of medication (Fig. 5). No AE leading to treatment discontinuation by APR was reported.

**Table 2:** Summary of AEs

AE	Ibandronate 0.5 mg	Ibandronate 1 mg	Risedronate
	(*n* = 411)	(*n* = 411)	(*n* = 406)
Any AE	406 (98.8 %)	401 (97.6 %)	393 (96.8 %)
Serious AEs	101 (24.6 %)	102 (24.8 %)	132 (32.5 %)
AEs leading to death	5 (1.2 %)	3 (0.7 %)	6 (1.5 %)
AEs leading to withdrawal	34 (8.3 %)	42 (10.2 %)	38 (9.4 %)
Serious AEs leading to withdrawal	27 (6.6 %)	28 (6.8 %)	27 (6.7 %)
Most common AEs			
Nasopharyngitis	188 (45.7 %)	209 (50.9 %)	201 (49.5 %)
Contusion	99 (24.1 %)	89 (21.7 %)	99 (24.4 %)
Osteoarthritis	75 (18.2 %)	63 (15.3 %)	51 (12.6 %)
Back pain	53 (12.9 %)	80 (19.5 %)	55 (13.5 %)
Arthralgia	54 (13.1 %)	47 (11.4 %)	38 (9.4 %)
Constipation	43 (10.5 %)	43 (10.5 %)	55 (13.5 %)
Diarrhea	23 (5.6 %)	15 (3.6 %)	19 (4.7 %)
Bronchitis	12 (2.9 %)	18 (4.4 %)	17 (4.2 %)
Urinary tract infection	9 (2.2 %)	4 (1.0 %)	10 (2.5 %)
Dyspepsia	13 (3.2 %)	11 (2.7 %)	12 (3.0 %)

*AE* adverse event

**Table 3:** AEs of interest

AE	Ibandronate 0.5 mg	Ibandronate 1 mg	Risedronate
	(*n* = 411)	(*n* = 411)	(*n* = 406)
Renal function related	12 (2.9 %)	11 (2.7 %)	8 (2.0 %)
GI related	113 (27.5 %)	120 (29.2 %)	108 (26.6 %)
Serious GI related	5 (1.2 %)	2 (0.5 %)	9 (2.2 %)
Cardiac related	7 (1.7 %)	5 (1.2 %)	4 (1.0 %)
APR related	37 (9.0 %)	46 (11.2 %)	20 (4.9 %)
Hypocalcemia	0	0	0
Osteonecrosis of the jaw^a^	0	0	0
Atypical fracture of the femur^a^	0	0	0

*AE* adverse event, *GI* gastrointestinal, *APR* acute phase reaction

^a^As per the American Society of Bone and Mineral Research case definition

## Discussion

We compared the efficacy and safety of IV ibandronate 0.5 mg and 1 mg/month with that of oral risedronate 2.5 mg daily (the licensed dose in Japan), in terms of fracture, BMD, and BTMs in postmenopausal women and older men with osteoporosis for registration purposes in Japan. Both doses of ibandronate were noninferior to risedronate with respect to the risk of vertebral fracture. As this active control study represents the first head-to-head comparison of the antifracture efficacy of two nitrogen-containing bisphosphonates, we closely mirrored the enrollment criteria and design of the registration trial of risedronate in Japanese patients with osteoporosis [19]. Indeed, the fracture rates observed with risedronate in our study compared well with those of the Japanese registration trial [19]. Significant increases in BMD from baseline were observed in all treatment groups, with ibandronate 1 mg demonstrating the greatest overall gains. Rapid decreases in BTMs were seen and were consistent with the BMD results. The safety profile of these agents was generally similar, and all regimens were well tolerated.

Over 3 years, the incidence of new or worsening vertebral fractures, and just new vertebral fractures, did not differ significantly between the ibandronate 0.5 and 1 mg groups, although fracture incidence was numerically higher with the 0.5 mg dose. The same findings were observed between the ibandronate 1 mg and risedronate groups, with a higher numerical incidence of fractures in the risedronate group. Both doses of ibandronate met the noninferiority criteria compared with risedronate. However, the HR for fracture incidence for 1 mg ibandronate versus risedronate was in fact smaller than for 0.5 mg ibandronate versus risedronate. Thus, the superiority of ibandronate over risedronate in increasing BMD may account for a small differential in fracture risk reduction [30]. Dose dependency of ibandronate on the incidence of vertebral fractures was shown numerically, but was not statistically significant. The efficacy of ibandronate in women only at 2 and 3 years was consistent.

We observed a similar rate of nonvertebral fractures between the ibandronate groups and the risedronate group, with the 1 mg ibandronate group having the lowest numerical incidence. The efficacy of 1 mg ibandronate was consistently greater than the 0.5 mg dose but this was not statistically significant. The 1 mg ibandronate group showed a nonsignificant improvement with a 27 % relative risk reduction in the incidence of the major six nonvertebral fractures versus risedronate. The VERT studies previously highlighted the nonvertebral fracture efficacy of risedronate in a white population [12, 13]. Additionally, a 3-year study in Japanese patients with osteoporosis who had previously undergone surgery for hip fracture, reported that risedronate 2.5 mg significantly reduced the incidence of contralateral hip fracture [20]. In the present study, the risk ratio of nonvertebral fracture between the 1 mg [annual cumulative exposure (ACE) 12 mg] and 0.5 mg (ACE 6 mg) ibandronate groups was 0.80 (95 % CI 0.47–1.36), which is within the range described in a pooled analysis of different ibandronate doses [31]. Our study therefore provides supportive data for the dose dependency of ibandronate on the risk of nonvertebral fractures in patients with osteoporosis.

BMD gains at all sites were substantial and significantly improved over baseline in all treatment groups. Treatment with IV ibandronate 1 mg/month in the current study resulted in similar BMD gains as obtained with IV ibandronate 2 mg/2 months and 3 mg quarterly in white women [1]. The effects of IV ibandronate on BMD gains appear to be dose-dependent and similar between Japanese and white patients. Changes in BTMs were comparable with those in previous studies and did not increase the risk of skeletal complications such as atypical femoral fractures over 3 years. Median changes from baseline in serum BALP in the risedronate 2.5 mg group were equivalent to changes observed with risedronate 5 mg in white patients [12, 13]. Compared with the 0.5 mg dose, treatment with IV ibandronate 1 mg/month resulted in significantly greater decreases in all BTMs. These data are compatible with the dose-dependent reductions in uCTX and serum OC reported with IV ibandronate 0.5 mg and 1 mg every 3 months [32]. Although the inhibition profile over time may differ between the dosing intervals of 1 and 3 months, the dose dependency on BTMs of IV ibandronate appears to be maintained. We noted similar reductions in uCTX and uNTX between the 1 mg ibandronate and risedronate groups in the present study, but reductions in serum BALP and OC differed between these groups, with greatest efficacy shown for 1 mg ibandronate [33]. Of note, the collection of urine and serum samples for BTM analysis was performed prior to monthly drug administration, at which point values were returning to baseline and the true effects of the time-course of treatment could not be seen.

The safety profile in this Japanese population was similar to previous studies in Western patients with no apparent increase in the nature and/or severity of AEs. APR was commonly experienced following the first administration of IV bisphosphonates. The range of symptoms that included specific (e.g., myalgia) and nonspecific (e.g., back pain, headache) AE terms were evaluated by onset and duration. The incidence of these symptoms was higher with ibandronate 1 mg than with oral risedronate, possibly due to the different administration routes, but they were reported at a similar frequency to previous studies [1, 9]. Symptoms defined as APR were mild to moderate in intensity, transient and associated with the first administration, as reported in earlier trials of ibandronate in osteoporosis [1, 4]. No difference in gastrointestinal, cardiac or renal AEs was noted among the treatment groups, possibly due to the double dummy design. Thus, 0.5 mg or 1 mg/month IV ibandronate appears to be well tolerated by Japanese osteoporotic patients with a similar safety profile to the established quarterly regimen in the Western population.

The study is limited by the lack of a placebo group due to ethical reasons for the 3-year treatment period in high-risk patients. In addition, the daily dose of supplemental vitamin D was low compared with recent studies in Western populations. Furthermore, differences in inclusion criteria existed in this study and the BONE and DIVA studies, such that the incidence rates of fracture and BMD values are not directly comparable.

In summary, monthly IV ibandronate demonstrated noninferiority to daily oral risedronate (at the dose licensed in Japan) in reducing the incidence of vertebral fractures in Japanese patients with primary osteoporosis. The safety profile of the 0.5 mg and 1 mg ibandronate doses and the 2.5 mg risedronate was comparable. These data suggest that 0.5 or 1 mg IV ibandronate is an effective option for the treatment of primary osteoporosis in Japanese patients, and that the 1 mg dose could be more beneficial in this patient group.
